# Enzyme Databases in the Era of Omics and Artificial Intelligence

**DOI:** 10.3390/ijms242316918

**Published:** 2023-11-29

**Authors:** Uroš Prešern, Marko Goličnik

**Affiliations:** Institute of Biochemistry and Molecular Genetics, Faculty of Medicine, University of Ljubljana, Vrazov trg 2, 1000 Ljubljana, Slovenia; uros.presern@mf.uni-lj.si

**Keywords:** enzyme, database, enzyme kinetics, standardization, BRENDA, SABIO-RK, MetaCyc, KEGG

## Abstract

Enzyme research is important for the development of various scientific fields such as medicine and biotechnology. Enzyme databases facilitate this research by providing a wide range of information relevant to research planning and data analysis. Over the years, various databases that cover different aspects of enzyme biology (e.g., kinetic parameters, enzyme occurrence, and reaction mechanisms) have been developed. Most of the databases are curated manually, which improves reliability of the information; however, such curation cannot keep pace with the exponential growth in published data. Lack of data standardization is another obstacle for data extraction and analysis. Improving machine readability of databases is especially important in the light of recent advances in deep learning algorithms that require big training datasets. This review provides information regarding the current state of enzyme databases, especially in relation to the ever-increasing amount of generated research data and recent advancements in artificial intelligence algorithms. Furthermore, it describes several enzyme databases, providing the reader with necessary information for their use.

## 1. Introduction

Enzymes represent a large and diverse group of biomolecules, catalyzing various chemical reactions in all living organisms. Although most enzymes are proteins, RNA molecules with catalytic functions (so-called ribozymes) exist as well [1]. Furthermore, synthetic deoxyribozymes have been designed [2], whereas natural deoxyribozymes remain to be discovered. Typically, enzymes represent 20–30% of the whole proteome of an organism, e.g., 22% of all human proteins possess some degree of catalytic function, whereas this proportion is higher for *Saccharomyces cerevisiae* (27%) and *Escherichia coli* (38%) (based on data from the protein database UniProtKB) [3]. Enzyme research spans over various research fields, from basic to applied life sciences, such as medical [4], pharmaceutical [5], agricultural [6], and biotechnological fields [7]. Information regarding the structure, function, distribution, and molecular and kinetic properties of enzymes is therefore essential to understand how enzymes work. This knowledge can be applied to the development of new drugs, diagnostics tools, green chemistry technologies, and food processing.

### 1.1. Enzyme Classification

**Figure 1:**
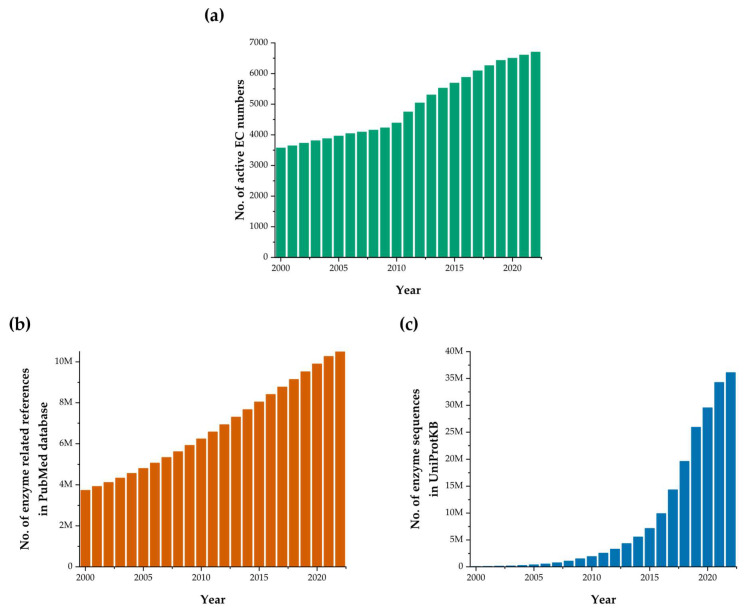
Growth of enzyme-related data in the last two decades. (**a**). The numbers of classified active entries (EC numbers) in the enzyme list. (**b**). The numbers of publications in PubMed that contain the terms *enzyme* or *metabolism*. (**c**) The numbers of protein sequences with annotated catalytic function in UniProtKB database.

The rapid rise in the number of newly discovered enzymes, which begun in the previous century, resulted in the need for systematic classification and nomenclature of enzymes based on their function and properties. For this purpose, the International Commission on Enzymes was established more than 50 years ago. The function of this initial commission is performed today by the Nomenclature Committee of the International Union of Biochemistry and Molecular Biology (NC-IUBMB). The International Commission introduced an enzyme classification system based on Enzyme Commission (EC) numbers associated with systematic and recommended enzyme names [8]. A four-level EC number is assigned to each enzyme based on its function, rather than its structure (e.g., EC 1.1.1.1 designates alcohol dehydrogenase). Consequently, all enzymes catalyzing the same reaction have the same EC number, even though their protein sequences differ and may not even be evolutionarily connected. The first digit of the EC number designates the class of an enzyme. There are seven classes: oxidoreductases, transferases, hydrolases, lyases, isomerases, ligases, and translocases, with translocases only added recently in 2018. The second and third digits of the EC number correspond to the subclass and sub-subclass, whereas the fourth digit represents a serial number. Currently, 6710 active EC numbers exist (with an additional 1346 EC numbers that were either deleted or transferred to a different number). Every year, approximately 100 new EC numbers are introduced (Figure 1a).

### 1.2. Enzyme Databases

In addition to the need for enzyme classification, a demand for systematic storage of an increasing amount of enzyme data (Figure 1b,c) arose as well. Enzyme databases serve as centralized repositories of information on enzymes, offering a wealth of data that can aid in various aspects of scientific research: gathering information, designing experiments, and performing comparative analyses, functional annotation, data integration, predictions, and modeling. Throughout the years, enzyme databases that cover different aspects of enzyme properties and functions have been established. The first and only overview of enzyme databases was published by Schomburg et al. in 2010 [9]. The functionalities of enzyme databases have improved since then and many new databases have been introduced. The aim of this article is therefore to present the updated state of the enzyme database field.

Enzyme databases can be classified as general databases, which cover all varieties of enzymes (Table 1), and specialized databases, which focus only on enzymes from a specific class or organism (Table S1). Certain enzyme databases provide only one type of information (e.g., enzyme nomenclature, kinetic parameters, reaction mechanisms, or involvement in metabolic pathways), whereas others are more comprehensive and gather diverse types of data. Additionally, enzyme data can be obtained from databases that are not specifically focused on enzymes per se but still contain relevant enzyme information (e.g., UniProt, Protein Data Bank (PDB), and Reactome). Corresponding data entries from different databases are usually cross-referenced, a fact which enables easier navigation among them. Secondary information resources such as Enzyme Portal offer a concise overview of enzyme data by integrating publicly available information from primary enzyme databases on one site [10].

## 2. The Quest to Standardize Data Reporting

The rise of the omics era has caused a huge increase in the amount of data gathered per experiment; however, incorporating all these data into databases has proven to be a challenging task. Most enzyme databases are manually curated, which is a time- and resource-consuming task. Research groups that manage such databases often have limited funding and therefore have troubles keeping databases up to date. Another obstacle during curation is encountering missing, incomplete, or ambiguous published data [39]. Exact information regarding experimental conditions is often lacking, which is problematic for data reproduction and analysis because enzyme properties and activity highly depend on parameters such as temperature, substrate/enzyme concentrations, and buffer composition. Analysis of published data has shown that 11%, 45%, 11%, and 22% of papers do not report data regarding temperature, enzyme concentration, substrate concentration, or buffer counterions, respectively [40]. Additionally, the lack of proper nomenclature and identifiers (e.g., EC numbers are provided in less than half of published papers) leads to ambiguous identification of used enzymes and chemical compounds [39]. Data relevant for inclusion in databases are often scattered throughout the text, graphs, and figures, a fact which prolongs the time needed for data curation [41]. Additionally, extracting data from graphs and figures does not always enable an accurate determination of parameters if the exact numerical value is not stated in the text.

Various efforts have been made to improve the quality and standardization of reported data. The development of ontologies and controlled vocabularies helps with problems regarding ambiguous data; however, implementing their use is hard to achieve in practice [39]. The initiative Standards for Reporting Enzyme Data (STRENDA) has been established to provide guidelines regarding which information should be included when reporting biocatalytic reactions and their results [42]. To date, more than 55 international biochemistry journals have adopted the STRENDA guidelines. To ensure datasets are complete and valid before submission, the STRENDA Database (STRENDA DB) was developed [17]. Data entered into the STRENDA DB are automatically checked for compliance with STRENDA guidelines, notifying the relevant researcher if required information is missing. When an article is published, its dataset, stored in the STRENDA DB, becomes public and is assigned a digital object identifier (DOI) for easier tracking. Such deposition of experimental data does not only ensure completeness of information but also simplifies the integration of these data into various enzyme databases, reducing the need for manual curation. To date, only 40 articles have submitted their data to the STRENDA DB since it was founded in 2017, signifying that the submission of experimental data has not yet become the norm.

EnzymeML represents another attempt to standardize data reporting and implement FAIR (findable, accessible, interoperable, reusable) principles of data management in the field of enzyme research [43]. EnzymeML is an extensible markup language-based data exchange format that enables the transfer of data among experimental platforms, modelling tools, and databases [44,45]. It comprises experimental data and metadata with implemented STRENDA guidelines. Original data (e.g., the time course of substrate concentrations) are also included, enabling other researchers to reanalyze experimental data. EnzymeML files can be uploaded to various repositories, such as Dataverse, and can be linked to the original research paper. Thus, data can be directly uploaded to enzyme databases, eliminating the need for manual curation.

## 3. The Future of Enzyme Databases in the Light of Recent Artificial Intelligence Developments

In recent years, scientific fields have been affected by the quick progress in artificial intelligence, and enzyme research is no exception. Although various prediction tools have already been present for several decades, deep-learning-based prediction models have considerably higher accuracy and precision. Deep learning algorithms for the prediction of enzyme function [46,47,48,49], structure [50,51], catalytic residues [52,53], and kinetic properties [54,55,56], as well as algorithms for the de novo design of enzymes [57,58], have already been developed.

Enzyme databases are important for the development of such algorithms because they contain useful information for the generation of training datasets. However, for databases to be used for this purpose, they must be machine-readable. While humans rely on context when selecting and interpreting data, the ability of computers to do the same is limited. Therefore, data must be organized in a systematic and predictable structure with a clear schema definition that describes how the data in a database are organized and how different parts relate to each other. Data should be presented in a consistent format with a standardized vocabulary to make it easier for machines to process information (a fact which underlines the usefulness of ontologies once again). It is also important that the data are accessible so that they can be easily imported into a data analysis tool [43]. Currently, most enzyme databases would benefit from improvements of their machine-readability, as they often do not present data in a sufficiently systematic way [18]. To this end, the standardization of data reporting (mentioned in the previous section) would help as well. Additionally, adjusting the architecture of databases to facilitate automatic data retrieval and analysis is necessary.

Apart from being used as a source for the training of datasets, enzyme databases can also be used for the deposition of predicted parameters generated by deep learning algorithms. A significant number of enzyme researchers lack the appropriate knowledge to run deep-learning-based software; therefore, such databases provide wider access to predicted data. Additionally, the acquisition of data is faster because the algorithm is run in advance [20].

This year has been marked by the breakthrough in the development of large language models (LLMs) such as GPT-4, BARD, and PaLM 2 [59]. LLMs are deep learning algorithms with a remarkable ability to understand and generate text-based content. Therefore, there is a great potential for the implementation of LLMs in the process of data extraction and curation in order to reduce the need for manual curation. This would vastly accelerate the speed of incorporation of new information into databases and, thus, alleviate the problem of maintaining databases up to date. Greater coverage of available experimental information would also improve subsequent data analysis and provide even larger training datasets for artificial intelligence prediction algorithms. In addition to data curation, LLMs could also be used for data analysis. Their ability to process complex queries could enhance search capabilities of databases in order to provide the user with the desired data. The usefulness of LLMs as prediction tools have also already been demonstrated [60].

## 4. Overview of General Enzyme Databases

The following section provides an overview of selected general enzyme databases. Data content, general architecture and properties, and available tools are presented for each database. In addition to the most widely used enzyme databases, GotEnzymes and TopEnzymes, which contain data generated by deep learning algorithms, are presented. Other specialized databases are listed in Table S1 in the Supplementary Material.

### 4.1. Enzyme Nomenclature Databases: ExplorEnz, IntEnz, and ExPASy ENZYME

ExplorEnz (https://www.enzyme-database.org/, accessed on 1 November 2023) is a primary source for IUBMB enzymes [12]. It contains basic data for all classified enzymes: IUBMB classification, accepted and systematic names as well as other names, catalyzed reaction, links to other databases, and references to the literature. It also provides guidelines and recommendations for enzyme naming. An input form enables researchers to report enzymes not yet classified or request changes to existing data.

Two additional enzyme classification databases are ExPASy ENZYME (https://enzyme.expasy.org/, accessed on 1 November 2023) [13] and IntEnz, Integrated relational Enzyme Database (https://www.ebi.ac.uk/intenz/, accessed on 1 November 2023) [14]. In comparison to ExplorEnz, they also provide enzyme sequence information by referencing corresponding data entries in UniProt. IntEnz additionally connects cofactor data to the Chemical Entities of Biological Interest database [61], thus improving standardization of its vocabulary.

### 4.2. BRENDA

BRENDA, Braunschweig Enzyme Database (www.brenda-enzymes.org, accessed on 1 November 2023), is a comprehensive enzyme information system [62]. Established in 1987, BRENDA started as a database containing manually curated enzyme-specific data extracted from the literature. However, other functionalities such as visualization tools, prediction algorithms, text mining methods, and integration from external sources were added throughout the years. It has been a part of the ELIXIR Core Data Resource since 2018 [63].

The database provides information on ~8400 enzyme entries (EC numbers). For enzymes not yet classified by NC-IUBMB, a preliminary BRENDA-supplied EC number is given. Each data entry is connected to its respective literature reference, source organism, and UniProt protein sequence ID, if available. Data entries are stored in ~50 categories covering enzyme classification and nomenclature, enzyme–ligand interactions, functional and kinetic parameters, molecular properties, stability, enzyme structure, organism-related information, isolation and preparation methods, application, related diseases, and references. Listed enzyme reactions include naturally occurring and synthetic substrates. Information regarding cofactors, inhibitors, and activating compounds is also included. All compounds that interact with specific enzymes are stored in the associated ‘BRENDA ligand database’ in which ligand structures are provided with their names and synonyms.

Importantly, BRENDA contains data on a wide spectrum of kinetic parameters (*K*_M_, *k*_cat_, *K*_i_, IC_50_, specific activity, temperature, and pH optimum/range). Numerical values of parameters are provided in standard units. Essential information describing experimental conditions used for determining parameters is described in the commentary section. This section is not fully standardized, which hinders automatic extraction and analysis of experimental conditions.

Each enzyme is presented alongside information regarding the organism source. In cases of multicellular organisms, the tissue type from which an enzyme originates is further specified. The terms used to describe the location and tissue type are based on the BRENDA Tissue Ontology [64]. To describe the subcellular localization, Gene Ontology terms are used [65,66]. Protein sequences and structures are retrieved from UniProt and PDB, respectively. Structures with active and binding sites can be visualized via an integrated NGL viewer [15,67].

The user can access information by performing text-based queries with quick, full-text, or advanced searches. The sequence search is useful for enzymes with known protein sequences. The Taxonomy Tree Explorer, EC Explorer, and Ontology Explorer can be used to search for enzymes in the taxonomic tree, hierarchical tree, and various biochemical ontologies, respectively. Enzyme ligands can be searched for with structure-based queries by drawing a chemical structure [63]. A substructure, isomer, or similarity search can be performed.

Text mining procedures complement manual annotation of data to provide complete overviews of the available literature. The data are derived from the titles and abstracts of all enzyme-related articles accessible within the PubMed database and are stored in the accessory repositories FRENDA, AMENDA, DRENDA, and KENDA. Data entries produced by text mining are classified into four categories based on their reliability level. FRENDA (Full Reference Enzyme Data) contains links to literature references providing information on enzyme occurrence in living organisms [68,69]. AMENDA (Automatic Mining of Enzyme Data) is a subset of FRENDA containing its most reliable data and additional information on the tissue source and subcellular localization of an enzyme [68,69]. While performing queries in BRENDA, one can choose whether to include data from FRENDA/AMENDA. DRENDA (Disease-Related Enzyme Information Database) contains information on enzyme–disease relations [70]. In addition to providing a reference link, these relations are classified into four categories: ‘Causal Interaction’, ‘Ongoing Research’, ‘Diagnostic Usage’, and ‘Therapeutic Application’. KENDA (Kinetic Enzyme Data) contains kinetic parameter data extracted from PubMed [70].

BRENDA employs visualization tools to offer clearer and easier access to enzyme data. A summary page of each enzyme class and organism (animal kingdom not yet included) contains a word cloud, offering a quick overview of the key aspects of the relevant research area [63,71]. The distribution of functional parameters can be visualized for a selected organism or enzyme class; however, this visualization is not interactive and does not have options to further specify a database query [67]. The BRENDA Genome Explorer provides a connection between genomic and enzymatic data. It displays enzyme genes in a selected genome and enables comparisons among genomic environments of an enzyme gene in different organisms [68]. BRENDA pathway maps visually display metabolic pathways with their associated enzymes and ligands [72]. Furthermore, users can upload their own transcriptomic, proteomic, or metabolomic data to analyze them in their metabolic context [15].

Prediction tools integrated in BRENDA enable additional annotation of enzyme data. EnzymeDetector provides genome-wide enzyme function annotation, which is performed by combining data from the main annotation databases and results in prediction algorithms that rely on sequence similarity analysis and sequence pattern searches [73]. Confidence scores are calculated based on the agreement level of different sources, thereby improving reliability and decreasing the error rate compared to using a single annotation source. Furthermore, implementation of TransMembrane Hidden Markov Model [74] and Target-P 1.1 [75] enables the prediction of the presence of transmembrane helices and subcellular localization of enzymes, respectively. The performances of these algorithms have been surpassed by deep-learning-based models [76,77], which have not yet been included in the BRENDA database.

BRENDA represents one of the most comprehensive databases for biochemical reactions, although it remains incomplete. The BKMS-react module (https://bkms.brenda-enzymes.org, accessed on 1 November 2023) combines the BRENDA database with three other main reaction databases: KEGG, MetaCyc, and SABIO-RK [29,63]. This provides a nonredundant combined list of biochemical reactions, enabling the creation of more accurate metabolic models.

All manually annotated BRENDA data can be downloaded as a single text file. Enzyme kinetic data can be exported in a Systems Biology Markup Language format as well [67]. Computer-based data acquisition is possible via the SOAP-based web service [69].

### 4.3. SABIO-RK

SABIO-RK, System for the Analysis of Biochemical Pathways—Reaction Kinetics (https://sabiork.h-its.org, accessed on 1 November 2023), is a manually curated database containing data on biochemical reactions and their kinetics [78,79]. Besides metabolic reactions, a small fraction of data consist of cellular signaling and transport reactions. Although the overall number of kinetic parameters in SABIO-RK is lower than that in BRENDA (e.g., ~51,000 *K*_M_ and ~28,000 *k*_cat_ values vs. ~177,000 *K*_M_ and ~87,000 *k*_cat_ values, respectively), SABIO-RK provides more structured and standardized data with separately stored experimental conditions and kinetic rate laws. This makes the extraction and analysis of kinetic data much easier compared to BRENDA, in which all experimental parameters are stored together in a commentary section in a non-standardized fashion with often incomplete information. Additionally, SABIO-RK enables more interactive visualization of kinetic data, facilitating searches and analyses [16].

In addition to manual data extraction from published literature by curators, data from laboratory experiments can also be submitted directly by users. Uploading EnzmyeML documents is possible, an option which simplifies submission of one’s own data [44]. At first, the selection of publications for data extraction was performed non-specifically by keyword searches in PubMed [79]. Nowadays, publications are selected based on SABIO-RK collaboration projects and user requests [16]. Consequently, certain organisms and enzyme classes are more represented than others.

Each data entry contains kinetic data of a single reaction performed under specific experimental conditions. If a publication contains data for multiple reactions, or the same reaction is performed under different experimental conditions, these data are stored in several distinct data entries. A data entry consists of a description of the biochemical reaction (substrates, products, enzyme class, organism taxonomy, and tissue and cellular location), kinetic data, and additional annotation data [80]. The type of kinetic model and associated equation are listed (e.g., Michaelis–Menten rate law, competitive inhibition, and Hill equation). The provided kinetic parameters include substrate and product concentrations and determined kinetic constants (e.g., *K*_m_, *k*_cat_, *V*_max_, *K*_I_, and Hill coefficient). Experimental conditions include pH, temperature, and buffer composition. Enzyme variants (wildtype, mutant, or recombinant) are specified as well. Any association of reactions with metabolic pathways and additional enzyme information are obtained from KEGG and UniProt. Standardization of the vocabulary used to enter the data is maintained using various ontologies (i.e., Chemical Entities of Biological Interest, BRENDA Tissue Ontology, Gene Ontology, Systems Biology Ontology, and NCBI taxonomy).

The database information can be queried with free text or advanced searches, and the results can be displayed as a list of individual data entries or grouped together based on corresponding reactions. SABIO-RK contains a tool for the interactive visualization of search results, which are presented in the form of heat maps, parallel coordinates, and scatter plots [16]. This provides a quick overview of the results and promotes a better understanding of the database content and connections among individual parameters. Kinetic parameter-based graphs enable the exploration of the kinetic data space and identification of outliers. By selecting data in the graphs, searches can be further refined, thus providing an alternative to ordinary text queries.

SABIO-RK data can be downloaded in a Systems Biology Markup Language format. Computer-based data acquisition is possible via RESTful web services.

### 4.4. Reaction Mechanism Databases: M-CSA and EzCatDB

M-CSA, Mechanism and Catalytic Site Atlas (https://www.ebi.ac.uk/thornton-srv/m-csa, accessed on 1 November 2023), is a manually curated database of enzyme reaction mechanisms and active sites [33]. It was built in 2017 by merging two separate databases: CSA—Catalytic Site Atlas [81] and MACiE—Mechanism, Annotation and Classification in Enzymes [82]. M-CSA provides information (position and role) on the catalytic residues, cofactors, and metal ions that participate in the catalyzation of a reaction as well as the annotation of overall reactions and their individual steps (if the mechanism is known). Reaction mechanisms are presented by curly arrow description, showing how electrons are transferred during each step of the reaction. A data entry in the database represents a unique reaction mechanism, meaning that two enzymes with identical mechanisms are not submitted as two separate entries (unless they are the result of convergent evolution). For each mechanism, a representative enzyme is chosen. Information regarding catalytic residue positions in the enzyme sequence and 3D structure are obtained from UniProt and PDB, respectively. Homologues of a representative enzyme are determined using PHMMER [83], and an analysis of catalytic residue conservation between homologues is provided.

Browsing and searching the data in M-CSA is possible in a text or graphical manner. Currently, the database contains 1003 entries, of which 734 contain detailed mechanistic descriptions. When M-CSA was set up in 2017, the existing data from CSA and MACiE were updated; however, inclusion of new data has been advancing slowly (58 new entries in the last 6 years).

EzCatDB, Enzyme Catalytic Mechanism Database (https://ezcatdb.cbrc.pj.aist.go.jp/EzCatDB, accessed on 1 November 2023), is a manually curated database that hierarchically classifies catalytic mechanisms based on four levels: basic reactions, ligand groups, catalytic mechanisms, and active sites [34]. Otherwise, it contains similar types of information as M-CSA, although it is slightly less comprehensive (880 entries).

### 4.5. MetaCyc

MetaCyc (https://metacyc.org/, accessed on 1 November 2023) is a manually curated database of metabolic pathways and enzymes [36]. It relies solely on experimental data and does not include computationally predicted pathways. MetaCyc contains information regarding metabolic pathways, reactions, compounds, enzymes, and their genes, with each having its own description page. However, all the information is interrelated, a fact which enables effective analysis of different relationships. Currently, it contains ~3100 pathways with ~18,500 reactions.

MetaCyc aims to represent true biological pathways without combining data from different organisms. If different variants of a pathway exist, they are recorded separately. Pathway data provide a list of reactions, background information on participating metabolites, relationships with respect to other pathways, taxonomic distribution, and references to relevant published literature. Information regarding taxonomic distribution of pathways is not comprehensive, and only organisms with well-studied pathways are included [84].

Enzyme data include molecular properties, kinetic parameters, tissue type, subcellular location, substrate specificity, and available experimental evidence. The name of the enzyme gene with a link to the external database containing sequence information is also provided.

The database can be searched via text-based queries (quick or advanced searches) or browsed using ontologies. BLAST searches using protein or nucleotide sequence are possible as well. MetaCyc includes a variety of specialized tools for visualization and analysis of metabolic networks [85,86,87]. Additionally, tools for the analysis of one’s own omics data are provided [88].

### 4.6. KEGG

KEGG, Kyoto Encyclopedia of Genes and Genomes (https://www.genome.jp/kegg/, accessed on 1 November 2023), is a composite database resource consisting of 16 manually curated databases that cover systems, genomic, chemical, and health information [35]. The KEGG Pathway database contains manually drawn reference maps, which reflect a union of experimentally determined biological pathways from multiple organisms (in contrast to MetaCyc, which provides only true biological pathways). Additionally, organism-specific pathways are generated computationally from genomic data using the KEGG Orthology system [89]. The KEGG Pathway database is linked with reaction and enzyme databases. In addition to NC-IUBMB classified reactions, additional reactions from the KEGG metabolic pathways are present. Currently, KEGG contains ~12,000 reactions. The KEGG Enzyme database contains information on enzyme substrates and products, links to reactions and pathways that the enzyme participates in, list of genes that code for that enzyme in different organisms, and references to the literature. KEGG enables text-based queries, searching via BLAST, and interactive browsing of metabolic maps and genomes. KEGG also contains tolls for analyses of omics data and functional characterization of genome sequences [90]; however, its array of tools is not as wide as that of MetaCyc [91].

### 4.7. Reactome

The Reactome Knowledgebase (https://reactome.org/, accessed on 1 November 2023) offers manually curated and peer-reviewed data on a wide range of biological processes [38]. In contrast to KEGG and MetaCyc, Reactome provides manual annotation only for human data. However, the semi-automatic identification of orthologous reactions enables the extension of the data to 15 non-human species.

The basic unit of Reactome is a reaction, which is defined as any event that converts inputs into outputs. Reactions in Reactome are therefore not limited to classical biochemical reactions, but also include ligand binding, complex formation and dissociation, conformational changes, etc. Physical entities that represent inputs and outputs of reactions can be of different types: e.g., small molecules, proteins, other macromolecules, and their complexes. Catalyzed reactions are associated with corresponding enzymes. Regulators (if present) are also linked to the reactions, together with information about their mode of action. Additionally, the reactions include information about the subcellular location and experimental evidence. Together with entities (inputs, outputs, enzymes, regulators), reactions are extensively cross-referenced with other databases and ontologies [92].

Series of reactions chained together by common inputs or outputs form pathways. Reactome currently contains ~2600 pathways and ~15,000 reactions involved in various cellular processes such as signal transduction, cell cycle, motility, and immune response. The annotation of pathogenic genomic variants, infectious pathogens genomic data, and molecular mechanisms of drug action enables the modeling of pathways in various diseases [93,94].

The Reactome database can be accessed with Pathway Browser, which enables the data to be searched via a graphical user interface. A full-text search is also possible. In addition, Reactome offers a range of tools for the analysis of experimental data. The protocols by Rothfels et al. provide instructions on how to use them [95].

### 4.8. GotEnzymes

GotEnzymes (https://metabolicatlas.org/gotenzymes, accessed on 1 November 2023) is a newly established database containing enzyme parameter predictions made by artificial intelligence [20]. It enables the retrieval of predicted parameters without the need for complete reproduction of a prediction pipeline (which can be resource-consuming). Such data can be used for statistical analysis and implementation into genome-scale metabolic models. The database currently contains predicted turnover numbers for 25.7 million enzyme–compound pairs from 8099 different organisms. The input data (protein sequences, compound structures, and EC numbers of reactions connecting enzymes with compounds) were extracted from KEGG, whereas predictions were made by the deep learning algorithm DLKcat [56]. Plans for future GotEnzymes releases comprise the inclusion of predictions for other kinetic parameters and implementation of annotations from other databases alongside KEGG.

### 4.9. TopEnzyme

TopEnzyme (https://cpclab.uni-duesseldorf.de/topenzyme, accessed on 1 November 2023) is a database of enzyme structure models containing more than 200,000 sequences [23]. Enzyme models are created with TopModel [51] and are linked to PDB, SWISS-MODEL repository [96], and AlphaFold Protein Structure Database [22]. Protein sequences used to generate models are extracted from the UniprotKB/Swiss-Prot database, thus covering 60% of all EC numbers. Although the AlphaFold Protein Structure Database provides models of unreviewed sequences in UniprotKB/TrEMBL as well, these are not included in TopEnzyme. Each data entry contains a Uniprot accession number and links to models and existing PDB structures. A confidence score for each model is provided. Additional information regarding enzyme name, function, EC number, organism source, and links to external databases is provided as well. Some structures also contain information regarding active and binding site residues.

## 5. Conclusions

Databases play an important role in enzyme research. To optimize the research process, constant improvements and upgrades of databases are necessary. Currently, one of the main focuses should be data standardization, which should be accomplished at the level of both data reporting in research articles and database curation. Although human-readability was the main goal of databases in the past, machine-readability has now become equally important, as it enables the performance of analyses of large amounts of data. Databases should also find a way to include data obtained from artificial intelligence prediction algorithms, which could close the gaps where experimental data are missing.

## Figures and Tables

**Table 1:** General enzyme databases and their properties. We retrieved the databases by searching PubMed for articles that contain the terms ‘database’, ‘repository’, or ‘resource’ in their title and the term ‘enzym*’ in their title or abstract. In addition, the Database Commons catalog [11] was searched for databases containing the keyword ‘enzym*’. Each recovered database was assessed with respect to whether it could be described as a general enzyme database and whether it was freely accessible and active. Only the databases that fulfil these criteria were included in the table.

Database Type	Database	Scope of Database	Data Source	Curation
Enzyme nomenclature	ExplorEnz	IUBMB classification [12]	IUBMB enzyme list	Manual
	ExPASy ENZYME	IUBMB classification with references to UniProt entries [13]	IUBMB enzyme list	Manual
	IntEnz	IUBMB classification with references to UniProt and ChEBI entries [14]	IUBMB enzyme list	Manual
Kinetics	BRENDA	Function and kinetic parameters, enzyme–ligand interactions, organism-related information, isolation methods [15]	Experimental (implementation of some prediction tools)	Manual and automated *
	SABIO-RK	Kinetic parameters with experimental conditions [16]	Experimental	Manual (option of data submission by experimenters)
	STRENDA-DB	Standardized kinetic data [17]	Experimental	Submission of data by experimenters
	IntEnzyDB	A comparison of kinetic parameters between wildtype and mutant enzymes [18]	Integration from multiple databases	Automated
	D3DistalMutation	Effects of mutations on enzyme activity [19]	Integration from multiple databases	Automated (mostly)
	GotEnzymes	Kinetic parameters predicted with a computer algorithm [20]	Predicted	Automated
Structure	UniProt	Protein sequence and functional information [3]	Experimental and predicted	Manual and automated
	PDB **	Experimentally verified protein structures [21]	Experimental	Manual
	AlphaFold DB **	Protein structures predicted with a computer algorithm [22]	Predicted	Automated
	TopEnzyme	Enzyme structures predicted with a computer algorithm [23]	Predicted	Automated
	Ligand-induced domain movements in enzymes	Data on movements of enzyme domains upon ligand binding [24]	Experimental	Manual and automated
	CoFactor	Data on organic enzyme cofactors [25]	Experimental	Manual and automated
	Natural Ligand DataBase	Structural data on enzyme–ligand interactions [26]	Experimental and predicted	Automated
Phylogeny	FunTree	Sequence, structural, and phylogenetic data on enzymes and other proteins fun [27]	Integration from multiple databases	Automated
Reactions (general)	ATLAS of Biochemistry	A database of all theoretical biochemical reactions [28]	Experimental and predicted	Automated
	BKMS-react	List of biochemical reactions from BRENDA, KEGG, MetaCyc, and SABIO-RK [29]	Integration from multiple databases	Automated
	EnzyMine	Mining of enzymatic reactions linked to sequence and structural annotations [30]	Integration from multiple databases	Manual
	Rhea	A resource of biochemical reactions [31]	IUBMB enzyme list	Manual
	Reaction explorer	Biochemical reactions derived from IUBMB enzyme list [32]	IUBMB enzyme list	Manual
Reaction mechanism	M-CSA	Information on position and role of catalytic residues and annotated step-by-step reaction mechanisms [33]	Experimental	Manual
	EzCatDB	A hierarchical classification of catalytic mechanisms [34]	Experimental	Manual
Metabolic pathways	KEGG	Information about metabolic pathways, reactions, metabolites, enzymes, and genes [35]	Experimental	Manual
	MetaCyc	Information about metabolic pathways, reactions, metabolites, enzymes, and genes [36]	Experimental	Manual
	PathBank	A metabolic pathway resource for model organisms [37]	Experimental	Manual
	Reactome	Information about biological pathways in human and model organisms [38]	Experimental	Manual and automated
Secondary information resource	Enzyme Portal	Integration of publicly available enzyme information [10]	Integration from multiple databases	Automated

* The BRENDA main repository is manually curated, while its accessory repositories are curated automatically. ** A general protein database covering proteins with or without catalytic function.

## Data Availability

No new data were created or analyzed in this study.

## Supplementary Materials

The following supporting information can be downloaded at: https://www.mdpi.com/article/10.3390/ijms242316918/s1. References [97,98,99,100,101,102,103,104,105,106,107,108,109,110,111,112,113,114,115,116,117,118,119,120,121,122,123,124,125,126,127,128,129,130,131,132,133,134,135,136,137,138,139,140,141,142,143,144,145,146,147,148,149,150,151,152,153,154,155,156,157,158,159,160,161,162,163,164,165,166,167,168,169,170,171,172,173,174,175,176] are listed in Supplementary Materials.
