# Supplementary material for: Enzyme Databases in the Era of Omics and Artificial Intelligence
Source: Int J Mol Sci. 2023 Nov 29;24(23):16918. doi: 10.3390/ijms242316918 (PMC10707154; doi:10.3390/ijms242316918)
Supplement: Supplementary file 1 [file ijms-24-16918-s001.zip › ijms-2726935-supplementary.pdf]

International Journal of *Molecular Sciences*

MDPI

Supplementary Material to

Enzyme databases in the era of omics and artificial intelligence

Table S1. Specialized enzyme databases. We retrieved the databases by searching PubMed for articles that contain terms *database*, *repository* or *resource* in their title and the term *enzym** in their title or abstract. In addition, the Database Commons catalog was searched for databases containing keyword *enzym**. Each database that was found was checked whether it could be described as a specialized enzyme database, and whether it was freely accessible and active. Only the databases that fulfil these criteria were included in the table.

Name	URL	Brief description
**Databases related to cytochromes P450**		
CYPedia	http://www-ibmp.u-strasbg.fr/~CYPedia/	Information about functional genomics of cytochromes P450 in *Arabidopsis thaliana* [1].
P450Rdb	https://www.cellknowledge.com.cn/p450rdb	A resource of cytochromes P450 and reactions catalyzed by them [2].
PCPD	https://p450.biodesign.ac.cn/	A repository of plant cytochromes P450 [3].
**Databases related to metabolic pathways and enzymes**		
ARALIP	http://aralip.plantbiology.msu.edu/	Information about metabolic pathways and enzymes involved in acyl-lipid metabolism in *A. thaliana* [4].
Database of Metabolic Enzymes Expressed in Kidney Tubule Segments	https://esbl.nhlbi.nih.gov/Databases/KSBP2/Targets/Lists/MetabolicEnzymes/	Information about expression levels of metabolic enzymes in different segments of kidney [5].
ECMDB	https://ecmdb.ca/	A metabolic pathway resource for *E. coli* [6].
HMDB	https://hmdb.ca/	A metabolic pathway resource for human [7].
MACADAM	https://macadam.toulouse.inra.fr/	Information about metabolic pathways and enzymes present in microbial taxonomic groups [8].
Mammalian Metabolic Enzyme Database	https://esbl.nhlbi.nih.gov/Databases/KSBP2/Targets/Lists/MetabolicEnzymes/MetabolicEnzymeDatabase.html	A catalog of mammalian metabolic enzymes [5].
PAMDB	http://pseudomonas.umaryland.edu/	A metabolic pathway resource for *Pseudomonas aeruginosa* [9].
PlantCyc	https://www.plantcyc.org/	A catalog of metabolic pathway databases of algal and plant genomes [10].
Trypanocyc	http://vm-trypanocyc.toulouse.inra.fr/	A metabolic pathway resource for *Trypanosoma brucei* [11].
YMDB	https://www.ymdb.ca/	A metabolic pathway resource for *S. cerevisiae* [12].
**Databases related to carbohydrate-active enzymes**		
CAZy	http://www.cazy.org	A family classification of carbohydrate-active enzymes [13].
CSDB_GT	http://csdb.glycoscience.ru/gt.html	A repository of experimentally confirmed glycosyltransferases from model organisms [14].
dbCAN-PUL	https://bcb.unl.edu/dbCAN_PUL/	A repository of prokaryotic gene clusters containing carbohydrate-active enzymes [15].
dbCAN-seq	https://bcb.unl.edu/dbCAN_seq/	Sequence and annotation data on carbohydrate-active enzymes and their gene clusters [16].
Galactosemia Proteins Database	http://www.protein-variants.eu/galactosemia/	Information about enzymes involved in the galactose metabolism and galactosemia [17].
MBPD	https://glycobio.org/	Information about enzymes involved in monosaccharide biosynthesis pathways [18].

PlantCAZyme	https://bcb.unl.edu/plantcazyme/	A repository of carbohydrate-active enzymes in plants [19].
**Databases concerning DNA- and RNA-related enzymes**		
CasPEDIA	http://caspedia.org/	A functional classification of class 2 Cas enzymes [20].
CasPDB	http://i.uestc.edu.cn/CASPDB/	A repository of bacterial and archaeal Cas enzymes [21].
DNAmoreDB	https://www.genesilico.pl/DNAmoreDB	Information about deoxyribozymes and their properties [22].
HEMD	https://mdl.shsmu.edu.cn/HEMD/	A repository of human epigenetic enzymes and chemical modulators [23].
LAHEDES	http://homingendonuclease.net/	Information about LAGLIDAG homing endonucleases and their target site specificity [24].
MODOMICS	https://genesilico.pl/modomics	Information about RNA modifications and RNA-modifying enzymes [25].
REBASE	http://rebase.neb.com/rebase/rebase.html	Information about restriction enzymes and DNA methyltransferases [26].
REPAIRtoire	https://repairtoire.genesilico.pl/	Information about DNA repair pathways and enzymes involved in them [27].
Ribocentre	https://www.ribocentre.org	Information about naturally occurring ribozymes and their properties [28].
RNAME	https://chenweilab.cn/rname/	A repository of RNA modification enzymes [29].
Telomerase database	https://telomerase.asu.edu/	Information about a telomerase enzyme and its substrates [30].
**Databases related to enzymes catalyzing post-translational modifications (PTM) and degradation of proteins**		
BrainPalmSeq	https://brainpalmseq.med.ubc.ca/	Information about expression of palmitoylating and depalmitoylating enzymes in the mouse brain [31].
CellPalmSeq	https://cellpalmseq.med.ubc.ca/	Information about expression of palmitoylating and depalmitoylating enzymes in human cells [32].
CaspBase	https://caspbase.uta.edu/index.html	A repository of caspase proteins [33].
DEPOD	https://depod.bioss.uni-freiburg.de	Information about human phosphatases and their substrates [34].
DUBs	https://esbl.nhlbi.nih.gov/Databases/KSBP2/Targets/Lists/DUBs/	A repository of mammalian deubiquitinating enzymes [35].
iPTMnet	https://research.bioinformatics.udel.edu/iptmnet/	Information about PTMs and PTM enzymes [36].
iUUCD	https://iuucd.biocuckoo.org	A systematic resource for ubiquitin and ubiquitin-related conjugates [37].
KinBase	http://kinase.com/web/current/kinbase	Comprehensive information about protein kinases from model organisms and human [38].
MEROPS	https://www.ebi.ac.uk/merops	Information about proteolytic enzymes, their inhibitors and substrates [39].
RESID	https://proteininformationresource.org/resid/	Information about PTMs and PTM enzymes [40].
SiMPLOD	http://fornerislab.unipv.it/SiMPLOD/	A collection of variants and mutants of collagen lysyl hydroxylases [41].
UbiNet 2.0	https://awi.cuhk.edu.cn/~ubinet/index.php	A repository of E3 ligase-substrate interactions [42].
**Databases related to enzyme-drug interactions**		
AECD	https://www.ceb.uminho.pt/aecd/	A resource for antimicrobial-enzyme combinations against *P. aeruginosa* and *Staphylococcus aureus* [43].
β-lactamase database	https://ifr48.timone.univ-mrs.fr/beta-lactamase/public/	Structural and functional information about β-lactamases [44].

BLDB	http://bldb.eu/	Structural and functional information about β-lactamases [45].
DrugMAP	http://drugmap.idrblab.net/	Information about drug molecules, their target proteins and drug-metabolizing enzymes [46].
INTEDE	http://intede.idrblab.net	Interactome of drug-metabolizing enzymes [47]
MagMD	http://119.3.41.228/MagMD/index.php	Information about interactions of gut microbes and their enzymes with drug molecules [48].
**Databases related to enzymes catalyzing degradation of plastics and xenobiotics**		
EAWAG-BBD	http://eawag-bbd.ethz.ch/index.html	A repository of microbial reactions and biodegradation pathways involving xenobiotics [49].
HADEG	https://github.com/jarojasva/HADEG	A repository of proteins and genes involved in hydrocarbon aerobic degradation [50].
OxDBase	http://crdd.osdd.net/raghava/oxdbase/	A repository of oxygenases involved in biodegradation of xenobiotics [51].
PAZy	https://www.pazy.eu	A repository of experimentally verified plastic-degrading enzymes [52].
PlasticDB	https://plasticdb.org/	A repository of plastic-degrading enzymes and organisms [53].
PMBD	http://pmbd.genome-mining.cn/home	Information about plastic-degrading enzymes and microorganisms [54].
RHObase	http://bicresources.jcbose.ac.in/ssaha4/Rhobase/	A repository of Rieske-type ring-hydroxylating oxygenases [55].
**Databases related to industrial use of enzymes and enzyme engineering**		
FEDA	https://feda.sciensano.be	Information related to food enzymes [56].
Glutantβase	http://bioinfo.dcc.ufmg.br/glutantbase/	Information about β-glucosidases [57].
GMEnzy	http://biotechlab.fudan.edu.cn/database/gmenzy	A repository of genetically modified enzymes with antibacterial properties [58].
IND-enzymes	https://indenzymes.srmist.edu.in/	A repository of hydrolytic enzymes from extremophilic bacterial species [59].
MetaBioME	http://metasystems.riken.jp/metabiome/	A curated database of commercially useful enzymes with an integrated platform for identification of their homologs [60].
ProtaBank	https://www.protabank.org	A repository for protein and enzyme design and engineering data [61].
RetroBioCat	https://retrobiocat.com/	Information on synthetic biotransformations [62].
RetroRules	https://retrorules.org/	A database of reaction rules for metabolic engineering [63].
**Other enzyme databases**		
B6 database	http://bioinformatics.unipr.it/B6db	Family classification of vitamin B6-dependent enzymes [64].
BioCatNet	https://www.biocatnet.de/	A catalog of family-specific enzyme databases [65].
ClusterCAD	https://clustercad.jbei.org/	A resource for assembly-line polyketide synthases and non-ribosomal peptide synthetases [66].
CyanoLyase	http://cyanolyase.genouest.org/	Information about sequences, motifs and functions of phycobilin lyases [67].
ESTHER Database	https://bioweb.supagro.inrae.fr/ESTHER/general?what=index	Family classification of proteins with alpha/beta-hydrolase fold [68].
HemeOxDB	http://www.researchdsf.unict.it/hemeoxdb	A repository of heme oxygenase inhibitors [69].
MeDBA	https://medba.ddtmlab.org/	Information about metalloenzymes and their ligands [70].

Orphan PKS catalog	https://orphanpkscatalog2022.stanford.edu	A catalog of assembly line polyketide synthase clusters [71].
PrenDB	http://prendb.pharmazie.uni-marburg.de/prendb/home/	A repository of prenyltransferases with an integrated substrate prediction tool [72].
ProtMiscuity	http://ufq.unq.edu.ar/protmiscuity	A database of enzymes with promiscuous catalytic activity [73].
RadicalSAM.org	https://radicalsam.org/	Sequence and functional data on enzymes belonging to radical SAM family [74].
RareLSD	https://webs.iiitd.edu.in/raghava/rarelsd/adv_lyso.php	A repository of lysosomal proteins associated with rare diseases [75].
RedoxiBase	https://peroxibase.toulouse.inra.fr	A phylogenomic overview of peroxidases and other ROS-related proteins [76].
SulfAtlas	https://sulfatlas.sb-roscoff.fr/	Family classification of sulfatases [77].
TeroKit	http://terokit.qmclab.com/	Information about terpenoid-derived molecules and enzymes involved in their biosynthesis [78].
ThYme	https://thyme.engr.unr.edu/v2.0	Family classification of enzymes acting on thioester-containing substrates [79].
TriForc database	http://bioinformatics.psb.ugent.be/triforc	A catalog of triterpene biosynthesis enzymes [80].

References

1. Ehlting J, Sauveplane V, Olry A, Ginglinger J-F, Provart NJ, Werck-Reichhart D (2008) An extensive (co-)expression analysis tool for the cytochrome P450 superfamily in Arabidopsis thaliana. BMC Plant Biol 8:47
2. Zhang Y, Pan X, Shi T, et al (2023) P450Rdb: A manually curated database of reactions catalyzed by cytochrome P450 enzymes. J Adv Res. https://doi.org/https://doi.org/10.1016/j.jare.2023.10.012
3. Wang H, Wang Q, Liu Y, et al (2021) PCPD: Plant cytochrome P450 database and web-based tools for structural construction and ligand docking. Synth Syst Biotechnol 6:102–109
4. Li-Beisson Y, Shorrosh B, Beisson F, et al (2013) Acyl-Lipid Metabolism. Arabidopsis Book. https://doi.org/10.1199/tab.0161
5. Corcoran CC, Grady CR, Pisitkun T, Parulekar J, Knepper MA (2016) From 20th century metabolic wall charts to 21st century systems biology: database of mammalian metabolic enzymes. Am J Physiol Renal Physiol 312:F533–F542
6. Sajed T, Marcu A, Ramirez M, Pon A, Guo AC, Knox C, Wilson M, Grant JR, Djoumbou Y, Wishart DS (2016) ECMDB 2.0: A richer resource for understanding the biochemistry of E. coli. Nucleic Acids Res 44:D495–D501
7. Wishart DS, Guo A, Oler E, et al (2022) HMDB 5.0: the Human Metabolome Database for 2022. Nucleic Acids Res 50:D622–D631
8. Le Boulch M, Déhais P, Combes S, Pascal G (2019) The MACADAM database: a MetAboliC pAthways DAtabase for Microbial taxonomic groups for mining potential metabolic capacities of archaeal and bacterial taxonomic groups. Database 2019:baz049
9. Huang W, Brewer LK, Jones JW, Nguyen AT, Marcu A, Wishart DS, Oglesby-Sherrouse AG, Kane MA, Wilks A (2018) PAMDB: a comprehensive Pseudomonas aeruginosa metabolome database. Nucleic Acids Res 46:D575–D580
10. Hawkins C, Ginzburg D, Zhao K, et al (2021) Plant Metabolic Network 15: A resource of genome-wide metabolism databases for 126 plants and algae. J Integr Plant Biol 63:1888–1905
11. Shameer S, Logan-Klumpler FJ, Vinson F, et al (2015) TrypanoCyc: a community-led biochemical pathways database for Trypanosoma brucei. Nucleic Acids Res 43:D637–D644
12. Ramirez-Gaona M, Marcu A, Pon A, Guo AC, Sajed T, Wishart NA, Karu N, Djoumbou Feunang Y, Arndt D, Wishart DS (2017) YMDB 2.0: a significantly expanded version of the yeast metabolome database. Nucleic Acids Res 45:D440–D445
13. Drula E, Garron M-L, Dogan S, Lombard V, Henrissat B, Terrapon N (2022) The carbohydrate-active enzyme database: functions and literature. Nucleic Acids Res 50:D571–D577
14. Egorova KS, Smirnova NS, Toukach P V (2021) CSDB_GT, a curated glycosyltransferase database with close-to-full coverage on three most studied nonanimal species. Glycobiology 31:524–529
15. Ausland C, Zheng J, Yi H, Yang B, Li T, Feng X, Zheng B, Yin Y (2021) dbCAN-PUL: a database of experimentally characterized CAZyme gene clusters and their substrates. Nucleic Acids Res 49:D523–D528
16. Zheng J, Hu B, Zhang X, Ge Q, Yan Y, Akresi J, Piyush V, Huang L, Yin Y (2023) dbCAN-seq update: CAZyme gene clusters and substrates in microbiomes. Nucleic Acids Res 51:D557–D563
17. d'Acierno A, Scafuri B, Facchiano A, Marabotti A (2018) The evolution of a Web resource: The Galactosemia Proteins Database 2.0. Hum Mutat 39:52–60
18. Srivastava J, Sunthar P, Balaji P V (2021) Monosaccharide biosynthesis pathways database. Glycobiology 31:1636–1644
19. Ekstrom A, Taujale R, McGinn N, Yin Y (2014) PlantCAZyme: a database for plant carbohydrate-active enzymes. Database 2014:bau079

20. Adler BA, Trinidad MI, Bellieny-Rabelo D, et al (2023) CasPEDIA Database: a functional classification system for class 2 CRISPR-Cas enzymes. Nucleic Acids Res gkad890
21. Tang Z, Chen S, Chen A, He B, Zhou Y, Chai G, Guo F, Huang J (2019) CasPDB: an integrated and annotated database for Cas proteins from bacteria and archaea. Database 2019:baz093
22. Ponce-Salvatierra A, Boccaletto P, Bujnicki JM (2021) DNAmoreDB, a database of DNAzymes. Nucleic Acids Res 49:D76–D81
23. Huang Z, Jiang H, Liu X, Chen Y, Wong J, Wang Q, Huang W, Shi T, Zhang J (2012) HEMD: An Integrated Tool of Human Epigenetic Enzymes and Chemical Modulators for Therapeutics. PLoS One 7:e39917-
24. Taylor GK, Petrucci LH, Lambert AR, Baxter SK, Jarjour J, Stoddard BL (2012) LAHEDES: the LAGLIDADG homing endonuclease database and engineering server. Nucleic Acids Res 40:W110–W116
25. Boccaletto P, Stefaniak F, Ray A, et al (2022) MODOMICS: a database of RNA modification pathways. 2021 update. Nucleic Acids Res 50:D231–D235
26. Roberts RJ, Vincze T, Posfai J, Macelis D (2023) REBASE: a database for DNA restriction and modification: enzymes, genes and genomes. Nucleic Acids Res 51:D629–D630
27. Milanowska K, Krwawicz J, Papaj G, Kosiński J, Poleszak K, Lesiak J, Osińska E, Rother K, Bujnicki JM (2011) REPAIRtoire—a database of DNA repair pathways. Nucleic Acids Res 39:D788–D792
28. Deng J, Shi Y, Peng X, et al (2023) Ribocentre: a database of ribozymes. Nucleic Acids Res 51:D262–D268
29. Nie F, Tang Q, Liu Y, Qin H, Liu S, Wu M, Feng P, Chen W (2022) RNAME: A comprehensive database of RNA modification enzymes. Comput Struct Biotechnol J 20:6244–6249
30. Podlevsky JD, Bley CJ, Omana R V, Qi X, Chen JJ-L (2008) The Telomerase Database. Nucleic Acids Res 36:D339–D343
31. Wild AR, Hogg PW, Flibotte S, Nasseri GG, Hollman RB, Abazari D, Haas K, Bamji SX (2022) Exploring the expression patterns of palmitoylating and de-palmitoylating enzymes in the mouse brain using the curated RNA-seq database BrainPalmSeq. Elife 11:e75804
32. Wild AR, Hogg PW, Flibotte S, Kochhar S, Hollman RB, Haas K, Bamji SX (2023) CellPalmSeq: A curated RNAseq database of palmitoylating and de-palmitoylating enzyme expression in human cell types and laboratory cell lines. Front Physiol 14:
33. Grinshpon RD, Williford A, Titus-McQuillan J, Clay Clark A (2018) The CaspBase: a curated database for evolutionary biochemical studies of caspase functional divergence and ancestral sequence inference. Protein Sci 27:1857–1870
34. Damle NP, Köhn M (2019) The human DEPhOsphorylation Database DEPOD: 2019 update. Database 2019:baz133
35. Xue Z, Chen J-X, Zhao Y, Medvar B, Knepper MA (2016) Data integration in physiology using Bayes' rule and minimum Bayes' factors: deubiquitylating enzymes in the renal collecting duct. Physiol Genomics 49:151–159
36. Huang H, Arighi CN, Ross KE, Ren J, Li G, Chen S-C, Wang Q, Cowart J, Vijay-Shanker K, Wu CH (2018) iPTMnet: an integrated resource for protein post-translational modification network discovery. Nucleic Acids Res 46:D542–D550
37. Zhou J, Xu Y, Lin S, Guo Y, Deng W, Zhang Y, Guo A, Xue Y (2018) iUUCD 2.0: an update with rich annotations for ubiquitin and ubiquitin-like conjugations. Nucleic Acids Res 46:D447–D453
38. Manning G, Whyte DB, Martinez R, Hunter T, Sudarsanam S (2002) The Protein Kinase Complement of the Human Genome. Science (1979) 298:1912–1934
39. Rawlings ND, Barrett AJ, Thomas PD, Huang X, Bateman A, Finn RD (2018) The MEROPS database of proteolytic enzymes, their substrates and inhibitors in 2017 and a comparison with peptidases in the PANTHER database. Nucleic Acids Res 46:D624–D632
40. Garavelli JS (2004) The RESID Database of Protein Modifications as a resource and annotation tool. Proteomics 4:1527–1533
41. Scietti L, Campioni M, Forneris F (2019) SiMPLOD, a Structure-Integrated Database of Collagen Lysyl Hydroxylase (LH/PLOD) Enzyme Variants. J Bone Miner Res 34:1376–1382
42. Li Z, Chen S, Jhong J-H, Pang Y, Huang K-Y, Li S, Lee T-Y (2021) UbiNet 2.0: a verified, classified, annotated and updated database of E3 ubiquitin ligase–substrate interactions. Database 2021:baab010
43. Jorge P, Alves D, Pereira MO (2019) Catalysing the way towards antimicrobial effectiveness: A systematic analysis and a new online resource for antimicrobial–enzyme combinations against Pseudomonas aeruginosa and Staphylococcus aureus. Int J Antimicrob Agents 53:598–605
44. Vivek K, M DS, Adrien E, Justine D, Olivier C, Laurent T, Jean-Marc R, Didier R, Pierre P (2019) An Integrative Database of β-Lactamase Enzymes: Sequences, Structures, Functions, and Phylogenetic Trees. Antimicrob Agents Chemother 63:10.1128/aac.02319-18
45. Naas T, Oueslati S, Bonnin RA, Dabos ML, Zavala A, Dortet L, Retailleau P, Iorga BI (2017) Beta-lactamase database (BLDB) – structure and function. J Enzyme Inhib Med Chem 32:917–919
46. Li F, Yin J, Lu M, et al (2023) DrugMAP: molecular atlas and pharma-information of all drugs. Nucleic Acids Res 51:D1288–D1299
47. Yin J, Li F, Zhou Y, et al (2021) INTEDE: interactome of drug-metabolizing enzymes. Nucleic Acids Res 49:D1233–D1243
48. Zhou J, Ouyang J, Gao Z, Qin H, Jun W, Shi T (2022) MagMD: Database summarizing the metabolic action of gut microbiota to drugs. Comput Struct Biotechnol J 20:6427–6430
49. Gao J, Ellis LBM, Wackett LP (2010) The University of Minnesota Biocatalysis/Biodegradation Database: improving public access. Nucleic Acids Res 38:D488–D491
50. Rojas-Vargas J, Castelán-Sánchez HG, Pardo-López L (2023) HADEG: A curated hydrocarbon aerobic degradation enzymes and genes database. Comput Biol Chem 107:107966

51. Arora PK, Kumar M, Chauhan A, Raghava GPS, Jain RK (2009) OxDBase: a database of oxygenases involved in biodegradation. BMC Res Notes 2:67
52. Buchholz PCF, Feuerriegel G, Zhang H, Perez-Garcia P, Nover L-L, Chow J, Streit WR, Pleiss J (2022) Plastics degradation by hydrolytic enzymes: The plastics-active enzymes database—PAZy. Proteins 90:1443–1456
53. Gambarini V, Pantos O, Kingsbury JM, Weaver L, Handley KM, Lear G (2022) PlasticDB: a database of microorganisms and proteins linked to plastic biodegradation. Database 2022:baac008
54. Gan Z, Zhang H (2019) PMBD: a Comprehensive Plastics Microbial Biodegradation Database. Database 2019:baz119
55. Chakraborty J, Jana T, Saha S, Dutta TK (2014) Ring-Hydroxylating Oxygenase database: a database of bacterial aromatic ring-hydroxylating oxygenases in the management of bioremediation and biocatalysis of aromatic compounds. Environ Microbiol Rep 6:519–523
56. Deckers M, Van Braeckel J, Vanneste K, Deforce D, Fraiture M-A, Roosens N h. c (2021) Food Enzyme Database (FEDA): a web application gathering information about food enzyme preparations available on the European market. Database 2021:baab060
57. Mariano D, Pantuza N, Santos LH, Rocha REO, de Lima LHF, Bleicher L, de Melo-Minardi RC (2020) Glutantβase: a database for improving the rational design of glucose-tolerant β-glucosidases. BMC Mol Cell Biol 21:50
58. Wu H, Huang J, Lu H, Li G, Huang Q (2014) GMEnzy: A Genetically Modified Enzybiotic Database. PLoS One 9:e103687-
59. Sunny JS, Nisha K, Natarajan A, Saleena LM (2021) IND-enzymes: a repository for hydrolytic enzymes derived from thermophilic and psychrophilic bacterial species with potential industrial usage. Extremophiles 25:319–325
60. Sharma VK, Kumar N, Prakash T, Taylor TD (2010) MetaBioME: a database to explore commercially useful enzymes in metagenomic datasets. Nucleic Acids Res 38:D468–D472
61. Wang CY, Chang PM, Ary ML, Allen BD, Chica RA, Mayo SL, Olafson BD (2018) ProtaBank: A repository for protein design and engineering data. Protein Sci 27:1113–1124
62. Finnigan W, Lubberink M, Hepworth LJ, et al (2023) RetroBioCat Database: A Platform for Collaborative Curation and Automated Meta-Analysis of Biocatalysis Data. ACS Catal 13:11771–11780
63. Duigou T, du Lac M, Carbonell P, Faulon J-L (2019) RetroRules: a database of reaction rules for engineering biology. Nucleic Acids Res 47:D1229–D1235
64. Percudani R, Peracchi A (2009) The B6 database: a tool for the description and classification of vitamin B6-dependent enzymatic activities and of the corresponding protein families. BMC Bioinformatics 10:273
65. Buchholz PCF, Vogel C, Reusch W, Pohl M, Rother D, Spieß AC, Pleiss J (2016) BioCatNet: A Database System for the Integration of Enzyme Sequences and Biocatalytic Experiments. ChemBioChem 17:2093–2098
66. Tao XB, LaFrance S, Xing Y, Nava AA, Martin HG, Keasling JD, Backman TWH (2023) ClusterCAD 2.0: an updated computational platform for chimeric type I polyketide synthase and nonribosomal peptide synthetase design. Nucleic Acids Res 51:D532–D538
67. Bretaudeau A, Coste F, Humily F, Garczarek L, Le Corguillé G, Six C, Ratin M, Collin O, Schluchter WM, Partensky F (2013) CyanoLyase: a database of phycobilin lyase sequences, motifs and functions. Nucleic Acids Res 41:D396–D401
68. Lenfant N, Hotelier T, Velluet E, Bourne Y, Marchot P, Chatonnet A (2013) ESTHER, the database of the α/β-hydrolase fold superfamily of proteins: tools to explore diversity of functions. Nucleic Acids Res 41:D423–D429
69. Amata E, Marrazzo A, Dichiara M, Modica MN, Salerno L, Prezzavento O, Nastasi G, Rescifina A, Romeo G, Pittalà V (2017) Heme Oxygenase Database (HemeOxDB) and QSAR Analysis of Isoform 1 Inhibitors. ChemMedChem 12:1873–1881
70. Yu J-L, Wu S, Zhou C, Dai Q-Q, Schofield CJ, Li G-B (2023) MeDBA: the Metalloenzyme Data Bank and Analysis platform. Nucleic Acids Res 51:D593–D602
71. Kishore S, Khosla C (2023) Genomic mining and diversity of assembly line polyketide synthases. Open Biol 13:230096
72. Gunera J, Kindinger F, Li S-M, Kolb P (2017) PrenDB, a Substrate Prediction Database to Enable Biocatalytic Use of Prenyltransferases * . J Biol Chem 292:4003–4021
73. Velez Rueda AJ, Palopoli N, Zacarías M, Sommese LM, Parisi G (2019) ProtMiscuity: a database of promiscuous proteins. Database 2019:baz103
74. Oberg N, Precord TW, Mitchell DA, Gerlt JA (2022) RadicalSAM.org: A Resource to Interpret Sequence-Function Space and Discover New Radical SAM Enzyme Chemistry. ACS Bio & Med Chem Au 2:22–35
75. Akhter S, Kaur H, Agrawal P, Raghava GPS (2019) RareLSD: a manually curated database of lysosomal enzymes associated with rare diseases. Database 2019:baz112
76. Savelli B, Li Q, Webber M, Jemmat AM, Robitaille A, Zamocky M, Mathé C, Dunand C (2019) RedoxiBase: A database for ROS homeostasis regulated proteins. Redox Biol 26:101247
77. Stam M, Lelièvre P, Hoebeke M, Corre E, Barbeyron T, Michel G (2023) SulfAtlas, the sulfatase database: state of the art and new developments. Nucleic Acids Res 51:D647–D653
78. Chen N, Zhang R, Zeng T, Zhang X, Wu R (2023) Developing TeroENZ and TeroMAP modules for the terpenome research platform TeroKit. Database 2023:baad020
79. Caswell BT, de Carvalho CC, Nguyen H, Roy M, Nguyen T, Cantu DC (2022) Thioesterase enzyme families: Functions, structures, and mechanisms. Protein Sci 31:652–676
80. Miettinen K, Iñigo S, Kreft L, Pollier J, De Bo C, Botzki A, Coppens F, Bak S, Goossens A (2018) The TriForC database: a comprehensive up-to-date resource of plant triterpene biosynthesis. Nucleic Acids Res 46:D586–D594
